# Duplication of *hsp-110* Is Implicated in Differential Success of *Globodera* Species under Climate Change

**DOI:** 10.1093/molbev/msy132

**Published:** 2018-06-28

**Authors:** Laura M Jones, Sebastian Eves-van den Akker, Patricija van-Oosten Hawle, Howard J Atkinson, Peter E Urwin

**Affiliations:** 1 Center for Plant Sciences, Faculty of Biological Sciences, University of Leeds, Leeds, UK; 2 School of Biology, University of Cambridge, Cambridge, UK; 3 School of Molecular and Cell Biology and Astbury Centre for Structural Molecular Biology, Faculty of Biological Sciences, University of Leeds, Leeds, UK

**Keywords:** *Globodera*, *Caenorhabditis elegans*, *hsf-1*, heat shock proteins, thermotolerance, gene duplication

## Abstract

Managing the emergence and spread of crop pests and pathogens is essential for global food security. Understanding how organisms have adapted to their native climate is key to predicting the impact of climate change. The potato cyst nematodes *Globodera pallida* and *G. rostochiensis* are economically important plant pathogens that cause yield losses of up to 50% in potato. The two species have different thermal optima that may relate to differences in the altitude of their regions of origin in the Andes. Here, we demonstrate that juveniles of *G. pallida* are less able to recover from heat stress than those of *G. rostochiensis*. Genome-wide analysis revealed that while both *Globodera* species respond to heat stress by induction of various protective heat-inducible genes, *G. pallida* experiences heat stress at lower temperatures. We use *C. elegans* as a model to demonstrate the dependence of the heat stress response on expression of Heat Shock Factor-1 (HSF-1). Moreover, we show that *hsp-110* is induced by heat stress in *G. rostochiensis*, but not in the less thermotolerant *G. pallida*. Sequence analysis revealed that this gene and its promoter was duplicated in *G. rostochiensis* and acquired thermoregulatory properties. We show that *hsp-110* is required for recovery from acute thermal stress in both *C. elegans* and in *G. rostochiensis*. Our findings point towards an underlying molecular mechanism that allows the differential expansion of one species relative to another closely related species under current climate change scenarios. Similar mechanisms may be true of other invertebrate species with pest status.

## Introduction

Understanding how animals respond to temperature is key to predicting the consequences of climate change. Since ectotherms are unable to regulate their body temperature as effectively as endotherms they have adapted to develop and function within temperature ranges that are typical for their habitat. However, there is little information regarding the regulation of the thermal limits of a species or how they respond to a different temperature range. Terrestrial ectotherms are likely to face increased periods of heat stress as mean temperatures and temperature variability are predicted to increase over the next few decades (Kharin et al. 2007; Smith et al. 2015). On one hand, there is growing evidence that small aquatic ectotherms with shorter generation times are able to rapidly adapt to climate change. For example, the water flea (*Daphnia magna*) in the UK and oceanic phytoplankton in the Gulf of Cariaco, Venezuela, have adapted to an increase in temperature over several years by increasing their thermal niche (Geerts et al. 2015; Irwin et al. 2015). On the other hand, many organisms respond to climate change by altering their geographic range. Both vertebrate and invertebrate species have moved towards higher latitudes over a circa 25 year period, presumably following the thermal optima to which they are already adapted (Hickling et al. 2006; Chen et al. 2011). Analysis of 612 crop pests and pathogens established a global move poleward since the 1960s for some organisms but not for either of the potato cyst nematode (PCN) species *Globodera pallida* or *G. rostochiensis* in the Northern hemisphere (Bebber et al. 2013).

The PCN *G. pallida* and *G. rostochiensis* are major pests of the potato crop in cool-temperate areas of the world. PCN is indigenous to South America (Evans et al. 1975; Plantard et al. 2008) but has been introduced to Europe and subsequently is now found in all major potato growing regions of the world (https://www.cabi.org/isc/datasheet/27033 and http://www.cabi.org/isc/datasheet/27034, last accessed September 14, 2017) where it causes losses to potato harvests of up to 50% (Trudgill 1986). PCN are host-specific parasites that coevolved over 15–21 My with wild potato species (*Solanum* L. section Petota Dumort.) in the cool-temperate climate of the Andean highlands. The two species are estimated to have diverged ∼18 Ma and display a shift in thermal range (Plantard et al. 2008). *Globodera pallida* is adapted to high altitudes and is considered to have undergone an expansion northwards within the current day Peru, Ecuador and Columbia as the Andean chain rose in that region during the Miocene. *Globodera rostochiensis* is hypothesised to originate from where uplift of the paleo-Andes was less extreme and therefore the climate is slightly warmer. However, the boundaries of the native geographical ranges for the two species, both at recent and geological time scales, are uncertain (Oro et al. 2014).

Optimal hatching occurs at 13–25 °C for *G. pallida* and 15–27 °C for *G. rostochiensis*, respectively (Kaczmarek et al. 2014). Females are able to develop within a temperature range of 15–25 °C for both species but optimal development for *G. pallida* occurs at 15–17.5 °C whilst that for *G. rostochiensis* occurs at 17.5–22.5 °C (Jones et al. 2017). *Globodera pallida* is also less tolerant of a diurnal temperature increase in soil temperature, with females taking longer to resume egg production (Jones et al. 2017). In the absence of adaptation, increased soil temperatures associated with climate change are predicted to decrease the pest status of *G. pallida* but benefit *G. rostochiensis*, particularly in southern UK (Jones et al. 2017). The molecular mechanisms underlying the differential temperature response of the two *Globodera* species are unknown. Fortuitously, much of our understanding of the heat stress response in animals has been guided by research on the model nematode, *Caenorhabditis elegans*.

*Caenorhabditis elegans* is also able to grow and reproduce within a 15–26 °C temperature range and, in response to a rapid temperature increase, an ancient and highly conserved program of stress-inducible gene expression, dependent on the transcription factor 1 HSF-1 and the thermosensory circuit is triggered to restore cellular protein homeostasis (Lindquist and Craig 1988; Morimoto 1998; Prahlad et al. 2008). Induced chaperones play an important part in disaggregation, refolding or degradation of aggregated or damaged proteins. In animal cells disaggregation requires the HSC-70 chaperone system (HSP-70, HSP-40, and HSP-110) and the HSP-90 chaperone system (Rampelt et al. 2012; Machida et al. 2016). HSP-110 acts as a nucleotide exchange factor, releasing peptide substrate from HSP-70 in an ATP‐dependent manner (Dragovic et al. 2006). RNAi knockdown of *hsp-110* (C30C11.4) in *C. elegans* results in increased aggregation of proteins at 12 and 24 h following a 1 h heat stress and, together with the Hsp70 member *hsp-1, hsp-110* is required for normal lifespan following heat stress (Rampelt et al. 2012). Small heat shock proteins (HSP-20s) have also been associated with recovery from heat stress in *C. elegans* and mammals (Kourtis et al. 2012; Tang et al. 2013).

The availability of draft genome sequences for both *G. pallida* and *G. rostochiensis* (Cotton et al. 2014; Eves-van den Akker, Laetsch et al. 2016) enabled us to investigate genome-wide changes in gene expression during recovery from heat stress, based on the paradigm of *C. elegans*. Increased expression of Hsp20 genes at lower hatch temperatures in *G. pallida* compared with *G. rostochiensis* supports a lower optimum temperature for this species. Moreover, although Hsp20, Hsp40, Hsp70, and Hsp90 gene family members are induced by heat stress in both *Globodera* species, *hsp-110* is heat inducible only in the more thermotolerant species, *G. rostochiensis*. Sequence analysis revealed that *hsp-110* was recently duplicated in the *G. rostochiensis* lineage with one gene gaining heat shock elements in the promoter region. Using RNAi we found that expression of *hsp-110* is required for recovery from acute heat stress in *C. elegans*, and is dependent on *hsf-1*. Confirmation of this role for *hsp-110* during recovery from heat stress in *G. rostochiensis* has led us to hypothesise that the recent duplication of *hsp-110* in *G. rostochiensis* may underlie its predisposition to exploit climate change.

## Results

### J2 Stage of *G. rostochiensis* Recover Faster from Acute Thermal Stress Than *G. pallida*

**Fig. 1.**
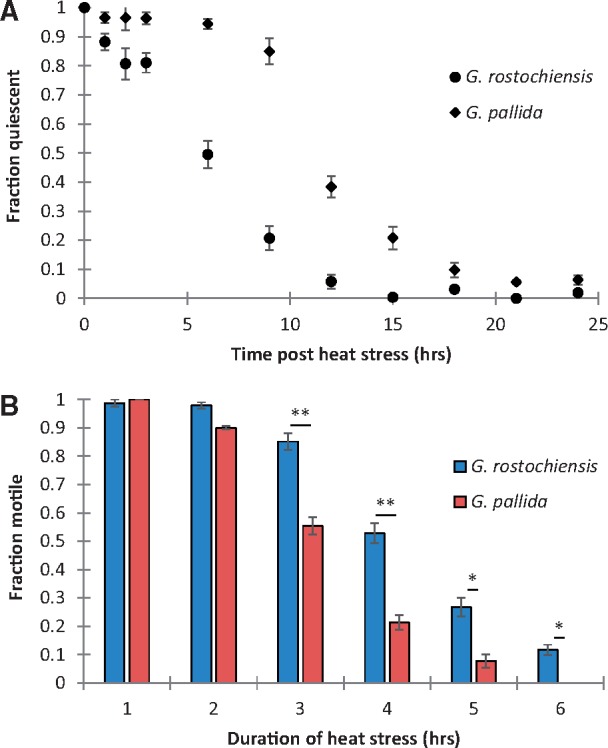
J2 stage of *G. rostochiensis* have a higher recovery from acute thermal stress than *G. pallida*. *G. rostochiensis* J2 stage has a lower rate of quiescence following a 1 h heat stress at 35 °C than *G. pallida* (*P* < 0.01) (*A*). Mean ± SEM, Log-rank (Mantel–Cox) test, *n* ≥ 5. *G. rostochiensis* J2 stage has a higher motility rate following a 35 °C heat stress for a duration of 3–6 h than *G. pallida* (*B*). Mean ± SEM, unpaired two-tailed Mann–Whitney test, *n* ≥ 5, *P < 0.05, **P < 0.01, ***P < 0.001.

We have previously shown that developing females of *G. pallida* take longer to recover from a prolonged diurnal heat stress than *G. rostochiensis* (Jones et al. 2017). To confirm that this phenomenon is life-stage independent, thus allowing us to study the underlying molecular basis in the technically more tractable infective juvenile, we exposed second-stage juveniles (J2s) to an acute heat stress (35 °C for 60 min) and measured the fraction motile every 1–3 h during a recovery period of 24 h. Immediately after heat stress, 100% of *G. pallida* and *G. rostochiensis* were quiescent, however, over the course of 24 h the more thermotolerant *G. rostochiensis* J2s recovered significantly faster than the less thermotolerant *G. pallida* (*P* < 0.01, fig. 1*a*). We also exposed second-stage juveniles (J2s) to an acute heat stress (35 °C for 0–6 h) and measured the fraction motile 24 h later. A significantly higher fraction of the more thermotolerant *G. rostochiensis* J2s was motile following a heat stress with a duration of 3–6 h than the less thermotolerant *G. pallida* (fig. 1*b*, *P* < 0.01 and *P* < 0.05).

### *G. pallida* has a Lower Thermal Limit Than *G. rostochiensis*

We investigated genome-wide gene expression profiles of both species following 60 min incubation at 20, 25, or 30 °C to explore the underlying molecular mechanisms for the lower ability of *G. pallida* to recover from heat stress. Forty-six and 60 transcripts were differentially expressed between conditions for *G. rostochiensis* and *G. pallida*, respectively (*P* < 0.001 and >2.5-fold, supplementary file S2, Supplementary Material online). Genome-wide gene expression at 20 and 25 °C are technically indistinguishable for each species and cluster together, separated from all three 30 °C biological replicates (supplementary fig. S1, Supplementary Material online). The majority of genes differentially expressed were upregulated at 30 °C compared with 20 and 25 °C: Following a 30 °C heat stress 37 transcripts were significantly upregulated in *G. rostochiensis* and 31 transcripts in *G. pallida* (*P* < 0.001 and >2.5-fold, supplementary file S2, Supplementary Material online). The regulation of 15 transcripts which were significantly upregulated in *G. rostochiensis* was confirmed by qPCR in both species (table 1, Pearson’s correlation = 0.973 and 0.923, *P* < 0.001 for *G. rostochiensis* and *G. pallida*, respectively). Within the top 12 most highly induced genes in both species were putative orthologues of hsp20, hsp40, hsp70, and hsp90 genes, which have roles in protein folding (GO: 0006457, GO: 0030968) and *gly-8* which is involved in protein glycosylation (GO: 0006486). The corresponding *C. elegans* orthologues are also significantly induced by heat stress (*P* < 0.001 and >2.5-fold [Brunquell et al. 2016]), demonstrating the suitability of *C. elegans* as a model to study the conserved heat stress response in plant-parasites.

**Table 1.** Members of Hsp20, Hsp40, Hsp70, and Hsp90 Gene Families Are Induced by Heat Stress in Both Species but Hsp110 Is Heat Inducible Only in *G. rostochiensis*.

Gene Name	*C. elegans* Homologue	*G. rostochiensis* 20 °C 1 h qPCR	*G. rostochiensis* 30 °C 1 h qPCR	*G. rostochiensis* Fold Change qPCR/ RNAseq		*G. pallida* 20 °C 1 h qPCR	*G. pallida* 30 °C 1 h qPCR	*G. pallida* Fold Change qPCR/ RNAseq	
GROS_g10683	*act-1/cdc-42*	1.00	1.00	1.00	1.02	1.00	1.00	1.00	1.09
GPLIN_001150200									
GROS_g02064	*hsp-16*	0.59 ± 0.06	15.97 ± 0.83	27.07	51.27	1.48 ± 0.07	12.75 ± 0.79	8.61	7.49
GPLIN_000903100	(hsp20)							
GROS_g09860	*hsp-16*	1.12 ± 0.10	19.07 ± 1.27	17.03	29.07	7.68 ± 0.92	40.79 ± 3.49	5.31	6.38
GPLIN_000680600	(hsp20)								
GROS_g10939	*hsp-16*	3.01 ± 0.36	48.42 ± 2.70	16.09	27.37	16.35 ± 2.41	97.37 ± 9.83	5.96	8.70
GPLIN_000021300	(hsp20)								
GROS_g14313	*hsp-16*	0.20 ± 0.03	1.78 ± 0.34	8.90	19.18	0.71 ± 0.06	4.59 ± 0.18	6.46	8.66
GPLIN_001518000	(hsp20)								
GROS_g03258	–	0.14 ± 0.02	0.94 ± 0.05	6.71	8.92	0.43 ± 0.05	1.45 ± 0.08	3.37	2.69
GPLIN_001642000									
GROS_g01391	*daf-21*	4.19 ± 0.21	30.62 ± 1.38	7.31	7.62	7.52 ± 0.69	35.80 ± 3.81	4.76	4.99
GPLIN_000887800	(hsp90)								
GROS_g14310	*hsp-1*	2.51 ± 0.70	21.30 ± 1.75	8.47	7.60	2.60 ± 0.13	15.95 ± 2.46	6.13	5.16
GPLIN_000080000	(hsp70)								
GROS_g11716	*hsp-16*	1.11 ± 0.12	9.53 ± 0.53	8.59	7.30	4.34 ± 0.41	21.74 ± 1.23	5.01	5.06
GPLIN_000159300	(hsp20)								
GROS_g05146	*hsp-16*	0.64 ± 0.11	2.09 ± 0.03	3.27	7.13	1.80 ± 0.31	3.05 ± 0.26	1.69	2.46
GPLIN_001642200	(hsp20)								
GROS_g05061	*gly-8*	0.24 ± 0.02	1.16 ± 0.06	4.83	7.05	1.09 ± 0.08	2.25 ± 0.14	2.06	2.85
GPLIN_001108000									
GROS_g02371	*hsp-110*	0.21 ± 0.02	0.86 ± 0.02	4.09	6.62	0.37 ± 0.01	0.36 ± 0.02	0.97	0.96
GPLIN_000265600	(hsp110)								
GROS_g04535	*dnj-13*	0.25 ± 0.04	1.09 ± 0.12	4.36	4.23	0.55 ± 0.04	1.48 ± 0.09	2.69	2.56
GPLIN_001642100	(hsp40)								
GROS_g04968	*ndk-1*	0.38 ± 0.03	1.06 ± 0.04	2.79	3.00	0.59 ± 0.06	1.26 ± 0.28	2.14	1.76
GPLIN_001010000									
GROS_g04417	*mnk-1*	0.04 ± 0.01	0.09 ± 0.03	2.25	2.88	0.37 ± 0.05	0.52 ± 0.04	1.41	1.40
GPLIN_000257900									
GROS_g13175	*kin-20*	0.09 ± 0.02	0.20 ± 0.01	2.22	2.86	0.20 ± 0.03	0.28 ± 0.01	1.40	1.80
GPLIN_000612600	(hsp20)								

Note.—Mean relative expression ± SEM following 20 °C for 1 h and 30 °C for 1 h from qPCR analysis, together with fold change from qPCR and RNAseq analysis for the 15 most heat inducible *Globodera* transcripts.

**Fig. 2.**
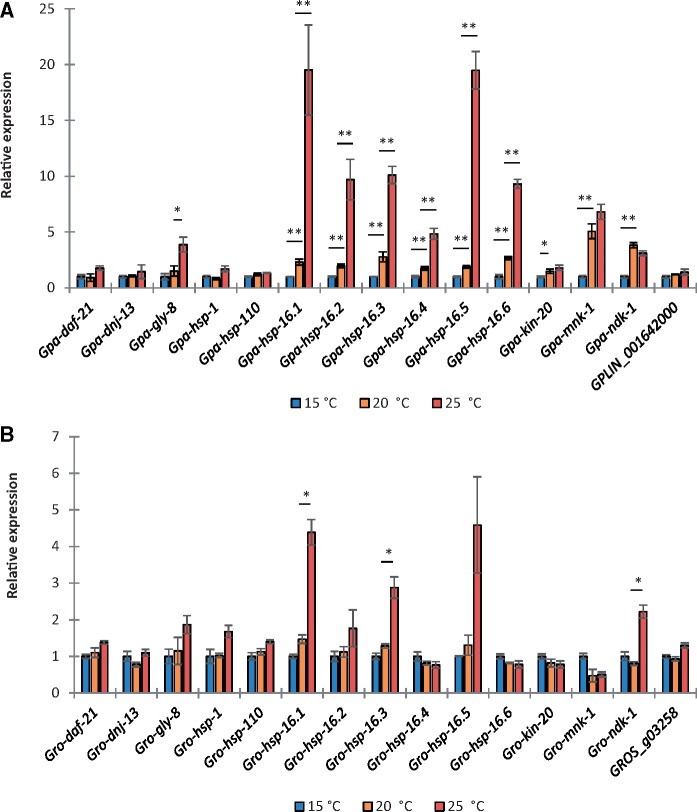
*G. pallida* has a lower thermal limit than *G. rostochiensis. G. pallida* has a significantly higher expression of hsp20 genes, *kin-20*, *mnk-1*, and *ndk-1* during hatch at 20 °C compared with 15 °C (*A*), unlike *G. rostochiensis* (*B*). Expression of other heat inducible genes is not significantly increased by hatch temperature in either species. Mean ± SEM, Kruskal–Wallis test and Dunn’s multiple comparison test, *n* ≥ 3, *P < 0.05, **P < 0.01, ***P < 0.001.

Interestingly, the expression of *mnk-1*, *ndk-1*, and *kin-20* homologues (which all have predicted roles in phosphorylation, GO: 0016310) was significantly (*P* < 0.001) increased by >2.5-fold following a 30 °C heat stress in the more thermotolerant *G. rostochiensis* but not in *G. pallida* (table 1)*.* Likewise, the six hsp20-like genes were also more strongly induced in *G. rostochiensis* than in *G. pallida*. This observation is likely due to an already higher relative expression of these genes in *G. pallida* J2s at the control temperature of 20 °C. Together with a reduced hatch rate and reduced female development for *G. pallida* at this temperature (Robinson et al. 1987; Jones et al. 2017), this observation prompted us to investigate the effect of hatch temperature (15, 20, and 25 °C) on gene expression. Although the expression of genes belonging to hsp40, hsp70, and hsp90 families did not significantly vary with hatch temperature in either species a significantly higher expression of all six hsp20 members, *mnk-1*, *ndk-1*, and *kin-20* was found when *G. pallida* were hatched at 20 °C or higher when compared with 15 °C (*P* < 0.05, fig. 2*a*). Increased expression of these genes was not observed in *G. rostochiensis* between 15 and 20 °C, although the expression of two hsp20 members and *ndk-1* did significantly increase between 20 and 25 °C (*P* < 0.05, fig. 2*b*), indicating activation of the heat stress response.

### Increased Expression of hsp20 Genes with Culture Temperature is *hsf-1*-dependent in *C. elegans*

**Fig. 3.**
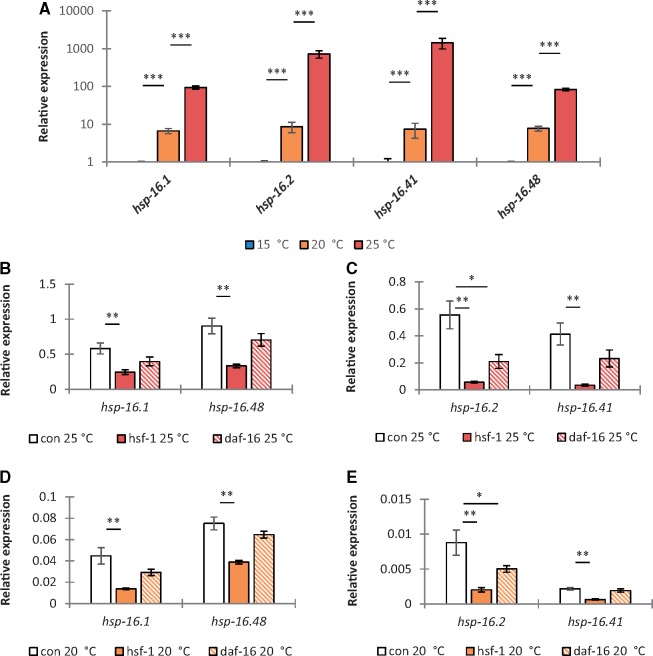
Increased expression of hsp20 genes with culture temperature is *hsf-1*-dependent in *C. elegans.* Expression of hsp20 genes in *C. elegans* is significantly higher during cultivation at 20 °C or 25 °C compared with 15 and 20 °C, respectively (*A*). Expression of hsp20 genes is significantly reduced under RNAi knockdown of *hsf-1* in *C. elegans* at 25 °C (*B* and *C*) and 20 °C (*D* and *E*) but not 15 °C (supplementary fig. S2, Supplementary Material online). Expression of *hsp-16.2* is also significantly reduced under RNAi knockdown of *daf-16* in *C. elegans* at 25 °C (*B* and *C*) and 20 °C (*D* and *E*) but not 15 °C (supplementary fig. S2, Supplementary Material online). Mean ± SEM, Kruskal–Wallis test with a Dunn’s multiple comparison test, *n* ≥ 3, *P < 0.05, **P < 0.01, ***P < 0.001.

In *C. elegans*, the expression of hsp20 members (but not *kin-20*, *mnk-1*, or *ndk-1*) also significantly increased with culture temperature between 15 and 20 °C (*P* < 0.05, fig. 3*a* and supplementary fig. S2*a*, Supplementary Material online), consistent with an optimal temperature of 18 °C for this species (Begasse et al. 2015; Gouvea et al. 2015). RNAi knockdown of the gene encoding Heat Shock Factor-1 (*hsf-1*) revealed that expression of this transcription factor is required for upregulation of *hsp-16.1*, *hsp-16.2*, *hsp-16.41*, and *hsp-16.48* with increased culture temperature in *C. elegans* (*P* < 0.01, fig. 3*b*–*e* and supplementary fig. S2*b* and *c*, Supplementary Material online). Assuming *hsf-1* is similarly conserved in *Globodera*, differential responses to heat stress described for *G. pallida* and *G. rostochiensis* could be explained by HSF-1 being active at a lower temperature in *G. pallida*. Interestingly, *daf-16* is also required for upregulation of *hsp-16.2* at higher temperatures in *C. elegans* (*P* < 0.05, fig. 3*b*–*e*) despite that the DAF-16 binding element (GTAAACA or TGTTTAC, (Furuyama et al. 2000) was apparently absent in the 2 kb region upstream of the four *hsp-16* genes.

### *hsp-110* is Heat Inducible in *G. rostochiensis* but not in *G. pallida*

**Fig. 4.**
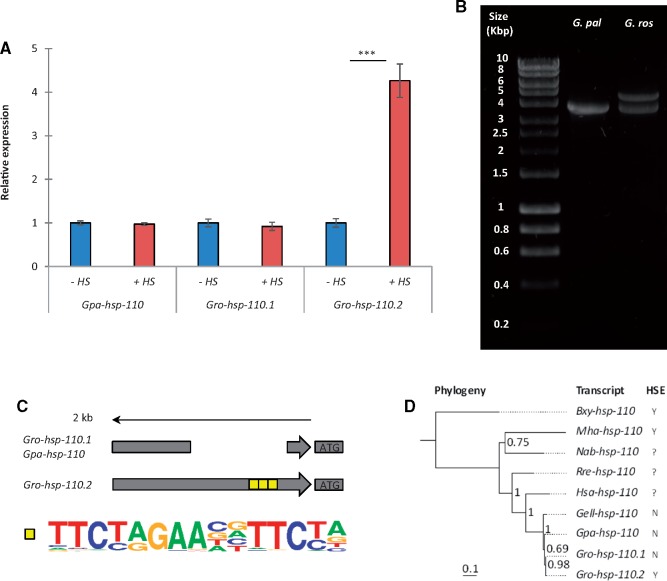
*Hsp-110* was recently duplicated and gained thermoregulation in *G. rostochiensis.* qPCR analyses reveals that the *hsp-110* transcript common to both *Globodera* species is not heat inducible, whereas the transcript unique to *G. rostochiensis* is heat inducible (*A*). Cloning and sequence analyses revealed that *hsp-110* and its promoter is duplicated in *G. rostochiensis* but only one copy is present in *G. pallida* (*B*), which is missing the heat shock element (*C*). Phylogenetic analysis with deduced amino acid sequences from *G. rostochiensis*, *G. pallida*, *G. ellingtonae*, *Heterodera sacchari*, *Rotylenchulus reniformis*, *Nacobbus aberrans*, *Meloidogyne hapla*, and *Bursaphelenchus xylophilus* reveals that duplication of *hsp-110* occurred relatively recently in the *G. rostochiensis* lineage. Identification of the heat shock element is indicated by present (Y), absent (N) or not known (?). Mean ± SEM, Kruskal–Wallis test with a Dunn’s multiple comparison test, *n* ≥ 3, *P < 0.05, **P < 0.01, ***P < 0.001.

Although a similar induction of genes encoding HSP-20, HSP-40, HSP-70, and HSP-90 chaperones was found in the two *Globodera* species, the HSP-70 nucleotide exchange factor encoded by *hsp-110* was heat inducible only in *G. rostochiensis* (table 1) and not in *G. pallida*. BLAST searching in the J2 transcriptome database for each species showed that two distinct *hsp-110* transcripts are present in *G. rostochiensis* and one in *G. pallida*, *Gro-hsp-110.1*, *Gro-hsp-110.2*, and *Gpa-hsp-110*, respectively (Cotton et al. 2014; Eves-van den Akker, Laetsch et al. 2016). The transcript unique to *G. rostochiensis* was three nucleotides longer and contained alterations in exons 14–16 (supplementary file S3, Supplementary Material online), allowing discriminatory qPCR analyses of the two transcripts. The unique transcript, *Gro-hsp-110.2* was induced by >4-fold in *G. rostochiensis* following heat stress (1 h at 35 °C), whereas the transcripts common to both species, *Gpa-hsp-110* and *Gro-hsp-110.1*, were not heat inducible (fig. 4*a*). Analysis of deduced amino acid sequences for both *G. rostochiensis* transcripts revealed that three of the nonsynonymous changes result in nonconservative amino acid substitutions, wheras a fourth alteration leads to the loss of five amino acids (supplementary fig. S3*a*, Supplementary Material online). However, alignment with amino acid sequence for *hsp-110* in yeast, for which the crystal structure has been solved (Liu and Hendrickson 2007), shows that the four nonconservative alterations occur within the flexible C-terminal region (Shaner et al. 2004) and are therefore unlikely to affect protein function (supplementary fig. S3*b*, Supplementary Material online).

### The *hsp-110* Gene Was Recently Duplicated in the *G. rostochiensis* Lineage

We determined gene copy numbers of *hsp-110* in *G. pallida* and *G. rostochiensis* by amplifying genomic fragments from each species using genus specific primers. We found that two copies of *hsp-110* are present in *G. rostochiensis* but only one in *G. pallida* (fig. 4*b* and *c*). BLAST searching in publicly available genomes, revealed that a single *hsp-110* gene is present in selected nematodes across the phylum (including *C. elegans*), and indeed the fruit fly and the mouse (supplementary table S1, Supplementary Material online). The two *G. rostochiensis hsp-110* paralogues are more similar to one another than they are to any other nematode sequence, indicative of a recent duplication (fig. 4*d*, supplementary file S4, Supplementary Material online). Consistent with a recent duplication in the *G. rostochiensis* lineage, phylogenetic analyses of *hsp-110* transcript sequences from *Globodera spp.*, *Heterodera sacchari*, *Rotylenchulus reniformis*, *Nacobbus aberrans*, *Bursaphelenchus xylophilus*, and *Meloidogyne hapla* separate the two *G. rostochiensis* sequences from all others in the phylogeny by a bootstrap support value of 0.98 (fig. 4*d*). Where genome sequence was available, we analysed the presence of canonical *C. elegans* heat shock elements (HSEs) in the promoter regions of all *hsp-110* genes in the phylogeny and representative species from other nematode clades, the fruit fly and mouse. At least one predicted HSE was found in the promoter region for all sequences outside the *Globodera*, indicating that they are likely heat inducible (supplementary table S1, Supplementary Material online). Within the *Globodera* species, only the recently duplicated *hsp-110* in *G. rostochiensis* contains predicted HSEs in its promoter, identified at −316, −334, and −374 bp from the start codon, within a 1,549 bp region which does not align to the original copy (fig. 4*c*, supplementary file S5, Supplementary Material online). Taken together, this suggests that the new *hsp-110* paralogue secondarily evolved to be heat responsive from an ancestral nonheat responsive *Globodera* gene.

### Expression of *hsp-110* is Required for Recovery from Acute Thermal Stress in Both *C. elegans* and *G. rostochiensis*

**Fig. 5.**
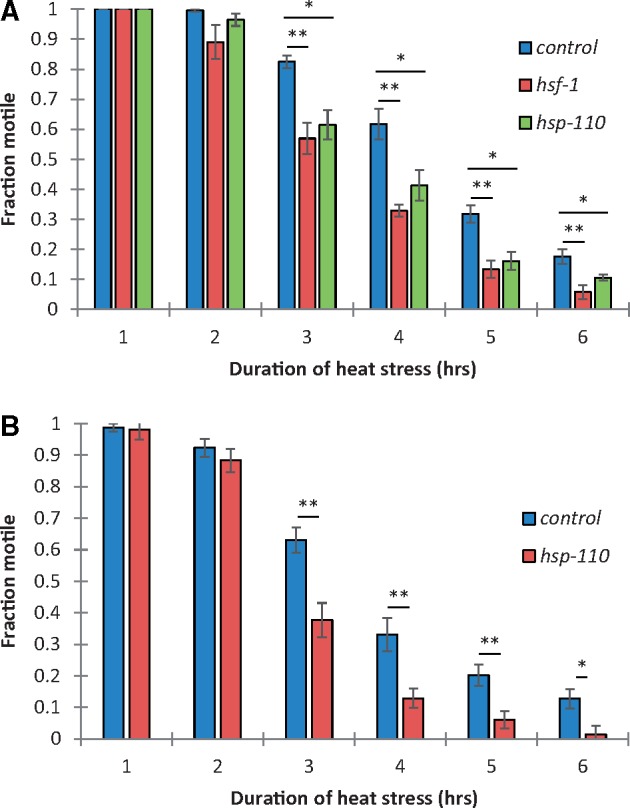
Expression of *hsp-110* is required for recovery from acute heat stress in *C. elegans* and *G. rostochiensis*. During RNAi knockdown of *hsp-110* recovery from a heat stress of 3–6 h duration was significantly reduced in *C. elegans* (*A*) and *G. rostochiensis* (*B*) compared with *gfp* controls. Mean ± SEM, unpaired two-tailed Mann–Whitney test, *n* ≥ 5, *P < 0.05, **P < 0.01, ***P < 0.001.

We show that *hsp-110* (C30C11.4) is indeed required for recovery from acute heat stress in *C. elegans* by assessing locomotion 24 h following a 35 °C heat stress during RNAi knockdown (fig. 5). This is consistent with a role for HSP-110 as a nucleotide exchange factor for the HSP-40-HSP70 disaggregation machinery and its requirement for normal lifespan following acute heat stress in *C. elegans* (Dragovic et al. 2006; Rampelt et al. 2012). RNAi knockdown of *hsf-1* or *hsp-110* (C30C11.4) in *C. elegans* resulted in a reduced recovery from acute heat stress, as indicated by a significantly lower fraction that were motile following a 3–6 h duration of 35 °C heat stress under RNAi knockdown of *hsf-1* or *hsp-110* (fig. 5*a*, *P* < 0.01 and *P* < 0.05, respectively). RNAi knockdown of *hsf-1* or *hsp-110* did not affect motility in the absence of heat stress and 100% nematodes were motile for all conditions. Measurement of *hsp-110* expression before and after heat stress indicated that *hsf-1* is required for normal induction of *hsp-110* following heat stress (supplementary fig. S4, Supplementary Material online, *P* < 0.01). Furthermore, by assessing locomotion of *G. rostochiensis* J2s during RNAi knockdown of *hsp-110* we confirm that this gene is also required for recovery from acute heat stress in this plant parasitic nematode (fig. 5*b*, *P* < 0.01). Due to the high sequence similarity of the two isoforms it was not possible to specifically target the heat inducible *hsp-110.2* but total *hsp-110* expression was significantly reduced by ∼86% with heat stress and ∼71% without heat stress (supplementary fig. S4*b*, Supplementary Material online, *P* < 0.01). This finding further supports a recent gene duplication of *hsp-110*, in the predisposition of *G. rostochiensis* to exploit climate change in the UK.

## Discussion

Understanding how animals have adapted to develop and function within temperature ranges that are typical for their habitat is key to predicting the effects of climate change. We recently showed that, without capacity for adaptation, climate change is likely to differently affect the pest status of the two species of *Globodera* in the UK as *G. pallida* has a lower thermal optimum for both hatching and subsequent development (Jones et al. 2017). Now we have strong evidence for some of the molecular mechanisms underlying this difference. We found that the heat stress response is triggered at a lower temperature in *G. pallida*, whilst the duplication of *hsp-110* and its gain of heat inducibility is implicated in the ability of *G. rostochiensis* to better withstand an acute heat shock.

Although expression of hsp20 small heat shock protein genes is induced by heat stress in both *Globodera* species, the particular temperature shift required for induction differs. For *G. rostochiensis*, significant increases in expression only occurred at 25 °C and above. Expression of these genes in *G. pallida* increased over the range 15–25 °C and was significantly higher at temperatures of 20 °C and above, consistent with an optimum temperature lower than 20 °C. A similar increase in expression of hsp20 genes with culture temperature was also found in *C. elegans*, consistent with an optimal temperature of 18 °C for this species (Begasse et al. 2015). RNAi knockdown revealed that increased induction of *hsp-16.1*, *hsp-16.2*, *hsp-16.41*, and *hsp-16.48* with culture temperature was dependent on *hsf-1* and that *daf-16* was also required for increased expression of *hsp-16.2* at higher temperatures. This is consistent with previous RNAseq analysis which found that *hsp-16* genes (but not members of hsp40, hsp70, and hsp90 gene families) were significantly down-regulated during RNAi knockdown of *hsf-1* in *C. elegans* cultured at 20 °C (Brunquell et al. 2016). Since the DAF-16 binding element (Furuyama et al. 2000) was not found in the promoter region of *hsp-16.2* it is possible that this gene is being regulated indirectly as previously suggested (Schuster et al. 2010). Although both HSF-1 and DAF-16 are required for survival at higher temperatures (i.e., thermotolerance) in *C. elegans* (Hsu et al. 2003; Walker et al. 2003; Hajdu-Cronin et al. 2004; Morley and Morimoto 2004; Lee and Kenyon 2009; Horikawa et al. 2015) a trade-off between activation of the heat stress response and fecundity has been reported (Casanueva et al. 2012; Aprison and Ruvinsky 2014; Labbadia and Morimoto 2015), acting as a “bet-hedging” strategy to diversify risk (Cohen 1966; Gillespie 1974; Balaban et al. 2004; Kussell and Leibler 2005). As HSF-1 can be directly activated by the neuronal circuit to pre-empt cellular damage caused by heat stress (Prahlad et al. 2008) it is likely that enhanced Hsp20 expression arises from adaptation of *G. pallida* to a lower thermal limit than *G. rostochiensis*.

The ability of *G. pallida* and *G. rostochiensis* to overcome potentially larger relative increases in warm extremes associated with increased temperature variability over land during the summer is also likely to differ (Gregory and Mitchell 1995; Kharin and Zwiers 2005). J2 stage of *G. pallida* are less thermotolerant than *G. rostochiensis*, taking significantly longer to recover from an acute thermal stress. This is consistent with our previous findings that females of *G. pallida* take significantly longer than those of *G. rostochiensis* to recover from a diurnal thermal stress (Jones et al. 2017). Transcriptomic analysis revealed that, although members of hsp40, hsp70, and hsp90 gene families are induced similarly in the two species by heat stress, interestingly *hsp-110* transcripts are heat inducible only in *G. rostochiensis.* Further investigation confirmed that this gene is duplicated in *G. rostochiensis* with the promoter of one gene copy harboring the HSEs that suggest regulation by the HSF-1 transcription factor (GuhaThakurta et al. 2002). Only a single copy of *hsp-110*, lacking HSEs, is present in *G. pallida* and the related species *G. ellingtonae*. Phylogenetic analysis suggests a relatively recent duplication of *hsp-110* and its promoter in the *G. rostochiensis* lineage during rapid expansion and diversification of *Globodera* species. Gene duplication events have been shown to produce rapid adaptive changes at the population level (Perry et al. 2007; Schrider and Hahn 2010; Bass and Field 2011) but divergence in protein function or expression is required for both copies to be maintained in the genome (Lynch and Conery 2003; Hahn 2009; Bass and Field 2011). Since nonconservative substitutions were not found within either predicted substrate- or nucleotide-binding domains, acquisition of HSEs in the promoter region, conferring enhanced expression and recovery from a thermal stress most likely explains the maintenance of two differently regulated copies of *hsp-110* in *G. rostochiensis*. A recent analysis of the Hsp40, Hsp70, and Hsp90 gene families in eusocial Hymenoptera which radiated across a wide range of thermal environments also found differences in gene copy number and in cis-regulation (Nguyen et al. 2016).

It was previously suggested that *G. pallida* became adapted to lower temperatures following the rise of the Andean chain during the Miocene, whereas *G. rostochiensis* originated from where uplift of the paleo-Andes was less extreme and therefore the climate was slightly warmer (Plantard et al. 2008). Our new analysis presents an alternative theory. It suggests that the three analysed *Globodera* species diverged from a common ancestor that was already adapted to a cooler climate. This ancestral species had presumably lost the thermoregulation of *hsp-110* found in other nematodes from all four clades (Supplementary Material online). The origins of *G. ellingtonae*, a species recently identified in three potato-growing fields within two US states, are unknown (Handoo et al. 2012). The presence in *G. ellingtonae* of only a single *hsp-110* gene lacking the promoter elements required for heat induction suggests, however, that both this species and *G. pallida* were adapted to a similar cool climate as their common ancestor. Presumably, during or after the speciation that gave rise to *G. rostochiensis*, movement to lower altitudes with a warmer climate provided selection pressure for the duplication and regain of heat regulation of *hsp-110*.

The requirement of *hsp-110* (C30C11.4) for recovery from acute heat stress in *C. elegans* is consistent with a role for HSP-110 as a nucleotide exchange factor for the HSP40-HSP70 disaggregation machinery and its necessity for normal lifespan following acute heat stress in *C. elegans* (Dragovic et al. 2006; Rampelt et al. 2012). It is likely that *hsp-110* is also required for maintaining protein homeostasis under physiological conditions since various developmental phenotypes of *C. elegans* under RNAi knockdown have also been reported (Gonczy et al. 2000; Kamath et al. 2003; Simmer et al. 2003; Sönnichsen et al. 2005). Constitutive expression has been observed in the pharynx, body wall muscle and nervous system of *C. elegans* (Dupuy et al. 2007). Similarly, hsp110 transcript and protein is present in most murine tissues and is highly expressed in the brain (Lee-Yoon et al. 1995; Yasuda et al. 1995). Although *hsp-110* is a single gene copy in mammals (Lee-Yoon et al. 1995), two isoforms have been identified (105α and 105β), which differ in their subcellular location and induction (Yasuda et al. 1995; Ishihara et al. 1999; Saito et al. 2009). The generally constitutive expression of Hsp105α has is increased by various stressors, whereas 105β is strictly heat inducible. The presence of these two differently regulated isoforms in mammals suggests that this gene is under high selection during both physiological and heat stress conditions.

An increase of up to 0.5 °C per decade has been projected for summer soil temperatures in the UK, with similar projected increases for sites in Sweden, Alaska and the northern forest (Houle et al. 2012; Jungqvist et al. 2014; Batir et al. 2017; Jones et al. 2017). Consequently, six out of seven English potato growing sites are predicted to have summer temperatures above the optimum temperature for hatch and female development of *G. pallida* (Robinson et al. 1987; Jones et al. 2017). Our analysis of the molecular mechanisms which underpin thermosensitivity in *Globodera* suggests that *G. pallida* has limited potential to alter its thermal limits within this context. Although numerous reports suggest that small aquatic ectotherms with short generation times are able to increase their thermal niche during climate change (Geerts et al. 2015; Irwin et al. 2015) the longer generation time makes it unlikely that *G. pallida* can increase its thermal limit for growth and reproduction at the rate of climate change. This is in agreement with lack of evidence for any terrestrial ectotherms with long generation times being able to adapt with climate change (Sinervo et al. 2010; Hoffmann and Sgro 2011; Scheffers et al. 2016). Although related species can differ by several degrees in their upper thermal limits, there is strong phylogenetic selection for upper limits (Hoffmann et al. 2013). If these reflect evolutionary constraints, substantial molecular changes (such as the duplication and expression divergence of genes) may be required to increase upper thermal limits. Since *G. rostochiensis* has the capacity to withstand higher soil temperatures, incorporation of qualitative resistance that is present in widely grown potato cultivars should be continued. Future control of *G. pallida* would be assisted by use of cultivars able to withstand climate change effects in the current potato growing areas.

## Materials and Methods

### *Globodera* Quiescence Assays

Second stage juveniles (J2) were hatched from encysted eggs of *G. pallida* Pa2/3 (population Lindley) or *G. rostochiensis* (pathotype Ro1) at 20 ± 0.5 °C using root diffusates collected from 3-week old potato plants. J2s were transferred by pipette into 1.5 ml high recovery tubes (Axygen) at a density of one juvenile per µl root diffusate in a volume of 100 µl. Juveniles were then incubated in a circulating water bath at 35 ± 0.5 °C for 60 min prior to being pipetted directly onto a 55 mm petri dish. After 1 min, motility was assessed over a 24-h period using a stereomicroscope. Each animal in the field of view at low magnification was observed for 3–4 s and scored as quiescent if they did not show an active sinusoidal form and movement. Approximately 30 nematodes were in the field of view and at least five observations of different worm batches (biological replicates) were used for each time point. Statistical analyses was carried out in GraphPad Prism version 7.02 using a log-rank (Mantel–Cox) test.

### *Globodera* Heat Stress Recovery Assays

Second stage juveniles (J2) were hatched from encysted eggs of *G. pallida* Pa2/3 (population Lindley) or *G. rostochiensis* (pathotype Ro1) at 20 ± 0.5 °C using root diffusates collected from 3-week old potato plants. J2s were transferred by pipette into 1.5 ml high recovery tubes (Axygen) at a density of one juvenile per µl root diffusate in a volume of 100 µl. Juveniles were then incubated in a circulating water bath at 35 ± 0.5 °C for 0–6 h prior to being placed on a rotating platform at 20 ± 0.5 °C to recover. Twenty-four hours later nematodes were pipetted directly onto a 55 mm petri dish and after 1 min, motility was assessed using a stereomicroscope. Each animal in the field of view at low magnification was observed for 3–4 s and scored as motile (recovered) if they showed detectable movement before or after gentle prodding with a platinum wire. Approximately 30 nematodes were in the field of view and at least five observations of different worm batches (biological replicates) were used for each time point. Statistical analyses were carried out in GraphPad Prism version 7.02 using an unpaired two-tailed Mann–Whitney test.

### Collection of *Globodera* J2s for RNAseq and qPCR

Cysts from *G. pallida* Pa2/3 (population Lindley) or *G. rostochiensis* (pathotype Ro1) were treated briefly with 1% sodium hypochlorite to release eggs as previously described (Cotton et al. 2014) that were allowed to hatch at 20 ± 0.5 °C in tap water (or at the appropriate temperature). After 5 days J2s were collected and pipetted into 1.5 ml high recovery tubes (Axygen) at a density of five juveniles per µl in tap water in a volume of 1 ml. Tubes were incubated, rotating, at 20, 25, or 30 ± 0.5 °C for 1 h. Nematodes were centrifuged at 100 × g for 30 s, residual water was aspirated and the remaining nematode pellets were flash frozen in liquid nitrogen. At least three biological replicates were used for each species and each incubation temperature.

### Growth of *C. elegans* during RNAi-Mediated Knockdown of *hsf-1* and *daf-16*

Wild-type N2 were grown from eggs at 15 ± 0.5 °C on NGM-LITE plates containing 50 µg ml^−1^ ampicillin, 12.5 µg ml^−1^ tetracycline and 1 mM isopropyl β-D-1-thiogalactopyranoside (IPTG), seeded with *Escherichia* coli HT115 (DE3) containing *pL4440:: gfp*, *pL4440:: hsf-1*, or *pL4440:: daf-16*. For each construct a 300–350 bp fragment was amplified from nematode cDNA using Platinum Taq DNA Polymerase High Fidelity (Invitrogen) and cloned into *pL4440* using BglII and KpnI restriction sites. Oligonucleotide sequences of the primers are given in supplementary file S1, Supplementary Material online. Ten adult hermaphrodites were transferred to fresh plates and allowed to lay embryos for 8 h before being removed. The progeny were allowed to continue growth to L4 stage at 15, 20, or 25 ± 0.5 °C prior to collection in M9 buffer. L4 stage nematodes were identified by the presence of a large white crescent-shaped mark in the vulval region (Koelle and Horvitz 1996). Nematodes were allowed to settle, residual M9 buffer was aspirated and the remaining nematode pellet was flash frozen in liquid nitrogen. At least three biological replicates were used for each RNAi condition and incubation temperature.

### *C. elegans* Heat Stress Recovery Assays

*C. elegans rrf-3*(pk1426) nematodes were grown on NGM-LITE plates containing 50 µg ml^−1^ ampicillin, 12.5 µg ml^−1^ tetracycline and 1 mM IPTG seeded with HT115 (DE3) containing *pL4440:: gfp*, *pL4440:: hsf-1*, or *pL4440:: C30C11.4*. For each construct 300–350 bp fragments were amplified from nematode cDNA using Platinum Taq DNA Polymerase High Fidelity (Invitrogen) and cloned into *pL4440* using BglII and KpnI restriction sites. Oligonucleotide sequences are given in supplementary file S1, Supplementary Material online. Mid L4-stage nematodes were identified by the presence of a white crescent-shaped mark in the vulval region (Koelle and Horvitz 1996), transferred to fresh plates and incubated in a standing incubator at 35 °C ± 0.5 °C for 0–6 h prior to being returned to 20 °C to recover. Motility was assessed 24 h later by direct observation using a stereomicroscope. Each animal in the field of view at low magnification was observed for 3–4 sec and scored as recovered if they showed detectable movement before or after gentle prodding with a platinum wire. Approximately 30 nematodes were in the field of view and at least five observations of different worm batches (biological replicates) were used for each time point. RNAi knockdown efficiency of *hsp-110* was assessed from approximately 30 nematodes. Statistical analyses were carried out in GraphPad Prism version 7.02 using a Kruskal Wallis test followed by Dunn’s multiple comparison test.

### RNAseq Analyses

Nematodes were disrupted in extraction buffer using a pestle motor mixer (Argos) and RNA was subsequently purified using an RNeasy mini kit (Qiagen). Quantity and quality of RNA was determined using a NanoDrop 2000 (Agilent) and 2100 BioAnalyser (Agilent). Libraries were prepared and 50 bp single end sequencing was carried out at the Next Generation Sequencing Facility at Leeds Institute of Biomedical and Clinical Sciences using an Illumina Hi Seq Instrument. Normalised gene expression values and differentially expressed genes were identified as previously described (Espada et al. 2016). In brief, raw reads were trimmed of adapter sequences and low quality bases (Phred <22, Trimmomatic [Bolger et al. 2014]), mapped to the genome (Tophat2, [Kim et al. 2013]), counted on a per gene basis (bedtools v2.16.2 [Quinlan and Hall 2010]), TMM normalised and differential expression analysis and clustering were performed using a Trinity wrapper pipeline and associated scripts for RSEM (Li and Dewey 2011) and EdgeR (Robinson et al. 2010) (FDR <0.001, minimum fold-change >2.5 [Haas et al. 2013]).

### qPCR Analyses in *Globodera* and *C. elegans*

RNA was extracted using an RNeasy kit (Qiagen) and cDNA was prepared using Superscript II Reverse Transcriptase (Invitrogen) from 500 ng RNA. Two stable transcripts (*act-1* and *cdc-42*) were used as normalising genes based on their previous use in *C. elegans* (Hoogewijs et al. 2008; Zhang et al. 2012) and unaltered transcript expression at different incubation temperatures from our RNAseq analysis in *Globodera* (*P* < 0.05). The oligonucleotide sequences of the primers are given in supplementary file S1, Supplementary Material online. SsoAdvanced™ Universal SYBR^®^ Green Supermix (Bio-Rad) was used without additional magnesium. The Bio-Rad CFX96 was programmed as follows; 30 s at 95 °C followed by 40 cycles of 15 s at 95 °C and 30 s at 60 °C. Raw data was analysed in Microsoft Excel and GraphPad Prism version 7.02 using a Kruskal–Wallis test followed by Dunn’s multiple comparison test.

### Cloning and Sequencing of *Globodera hsp-110*

The *hsp-110* gene and its promoter region (up to 2, 182 bp upstream of the start codon) was amplified from genomic DNA for each species using Platinum Taq DNA Polymerase High Fidelity (Invitrogen) and genus specific primers designed using the current genome assembly for both species (given in supplementary file S1, Supplementary Material online). A T100^TM^ Thermo Cycler (Bio-Rad) was programmed as follows; 3 min at 95 °C followed by 35 cycles of 30 s at 95 °C, 30 s at 55 °C and 5 min at 68 °C. PCR products were purified using the MicroElute cycle-pure kit (Omega) and cloned into pGEM T-easy (Promega) according to the manufacturer’s instructions. Plasmids were extracted using the QIAprep Spin miniprep kit (Qiagen) and sequencing was carried out by GATC Biotech using M13 sequencing primers and those given in supplementary file S1, Supplementary Material online. At least two clones for each construct were sequenced. Sequences were aligned using MUSCLE v3.8.3.1 (Edgar 2004).

### Identification of *hsp110* in Other Nematode/Metazoan Genomes

Hsp110 orthologues were identified by reciprocal BLAST searching (Altschul et al. 1990) using the human hsp110 NM_006644.3 in the genomes of *Globodera ellingtonae*, representative nematode species from other clades, the fruit fly and mouse (available at http://parasite.wormbase.org/Tools/Blast?db=core and https://blast.ncbi.nlm.nih.gov/Blast.cgi, last accessed September 21, 2017). Promoter regions (500 bp upstream of the start codon) were manually scanned for the heat shock element (HSE), previously defined as a binding site for *C. elegans* HSF-1 (GuhaThakurta et al. 2002).

### Phylogenetic Analysis for *hsp110* in Plant Parasitic Nematodes

Phylogenetic analyses were carried out using deduced amino acid sequences of *hsp-110* transcripts from the transcriptomes of *G. rostochiensis* (Eves-van den Akker, Laetsch et al. 2016), *G. pallida* (Cotton et al. 2014), *G. ellingtonae* (Phillips et al. 2017), *Heterodera sacchari* (Eves-van den Akker, personal communication, Sept 2017), *Rotylenchulus reniformis* (Eves-van den Akker, Lilley et al. 2016), and *Nacobbus aberrans* (Eves-van den Akker et al. 2014) along with those of predicted transcript sequences for *Meloidogyne hapla* (Opperman et al. 2008) and *Bursaphelenchus xylophilus* (Kikuchi et al. 2011). Sequences were aligned and refined using MUSCLE v3.8.3.1 (Edgar 2004). The alignment was trimmed using TrimAL (-gappyout) (Capella-Gutiérrez et al. 2009) and subject to model selection (JTT+GAMMA) and Bayesian phylogeny construction (Mr Bayes) with 100,000 generations and a burn in rate of 30% was carried out in TOPALi v2.5 (Milne et al. 2009). The phylogeny was out-group rooted by the *B. xylophilus* sequence (Blaxter et al. 1998) using FigTree v1.4 (http://tree.bio.ed.ac.uk/software/figtree/).

## Supplementary Material

Supplementary Data
