# Supplementary material for: Duplication of hsp-110 Is Implicated in Differential Success of Globodera Species under Climate Change
Source: Mol Biol Evol. 2018 Jun 28;35(10):2401–13. doi: 10.1093/molbev/msy132 (PMC6188557; doi:10.1093/molbev/msy132)
Supplement: Supplementary Data [file msy132_supp.zip › Supplementary file 1.pdf]

Supplementary file 1

Table S1. A single heat inducible copy of *hsp-110* was identified in all species except for *G. rostochiensis*. Hsp110 was identified by reciprocal BLAST searching in available genomes for the closely related *Globodera ellingtonae*, representative nematode species from other clades, the fruit fly and mouse. Location of the predicted heat shock element (HSE) from the first codon is also given.

Metazoan Genome	Description	hsp110 homologue	Predicted HSE from start codon
Trichinella spiralis (Mitreva *et al.*, 2011)	Clade I nematode	HSPA4L	-11,-101
Brugia malayi (Ghedin *et al.*, 2007)	Clade III nematode	Bma-hsp-110	-204
Loa loa (Aarnio *et al.*, 2014)	Clade III nematode	LOAG_07269	-228
Bursaphelenchus xylophilus (Kikuchi *et al.*, 2011)	Clade IV nematode	BXY_1161100.1	-216
Globodera pallida (Cotton *et al.*, 2014)	Clade IV nematode	GPLIN_000265600	-
Globodera rostochiensis (Eves-van den Akker *et al.*, 2016a)	Clade IV nematode	GROS_g02371	-316, -334, -374
Globodera ellingtonae (Phillips *et al.*, 2017b)	Clade IV nematode	g635.t1	-
Meloidogyne hapla (Opperman *et al.*, 2008)	Clade IV nematode	MhA1_Contig2367.frz3.fgene1	-116, -128
Caenorhabditis elegans (Consortium, 1998)	Clade V nematode	C30C11.4	-232, -135
Haemonchus contortus (Schwarz *et al.*, 2013)	Clade V nematode	augustus-scaffold11576-abinit-gene-0.0-mRNA-1	-75 -179
Necator americanus (Tang *et al.*, 2014)	Clade V nematode	NECAME_02547	-405
Drosophila melanogaster (Adams *et al.*, 2000)	Fruit fly	NP_648687.1	-124
Mus musculus (Mouse Genome Sequencing *et al.*, 2002)	Mouse	ENSMUSP00000144413	-64, -128

Figure S1

A)

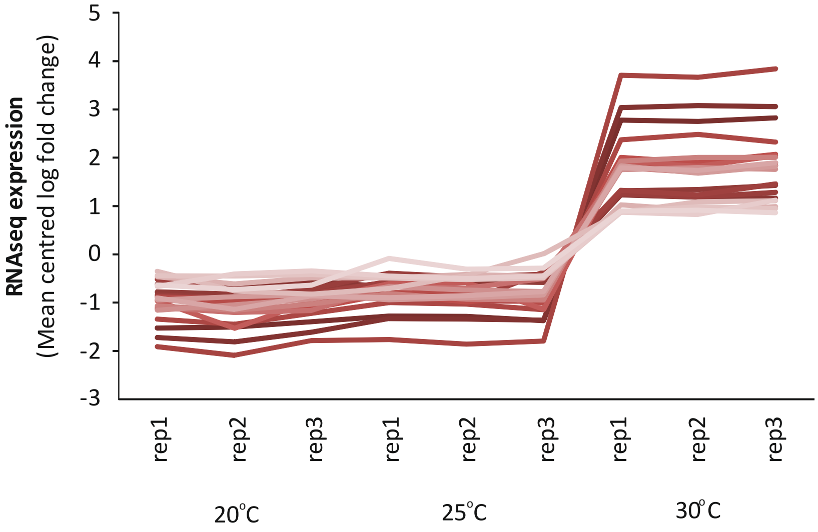

B)

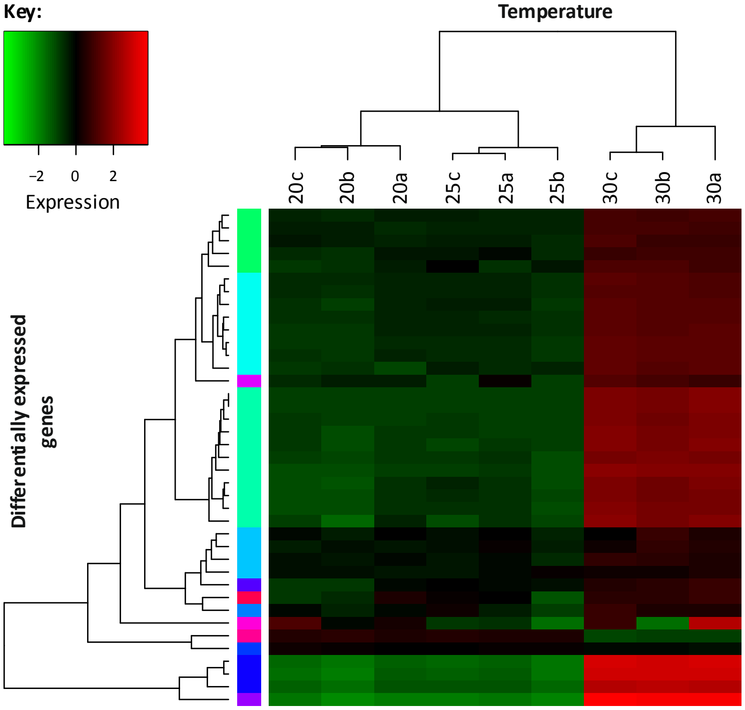

Figure S1. Gene expression in *Globodera* was significantly changed following a heat stress for 1 hr at 30 °C but not at 25 °C. Fold-change of transcript expression for 39 genes which had a significantly different expression following a 1hr heat stress at 30 °C in *G. rostochiensis* (p < 0.01, > 2.5 fold change) but not at 25 °C, represented by a line graph (A) and a heat map (B).

Figure S2.

A)

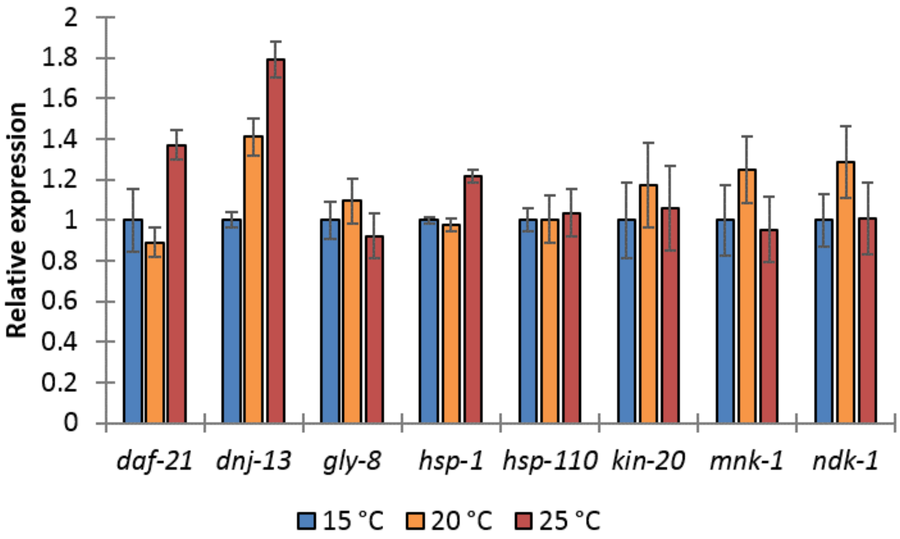

B)

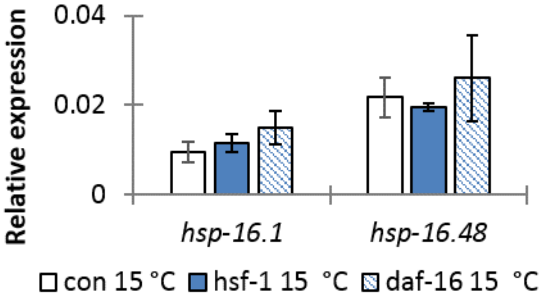

C)

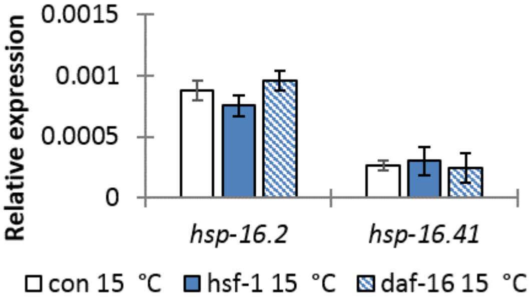

Figure S2. Increased expression of hsp20 genes with culture temperature is *hsf-1*- dependent in *C. elegans*. Expression of other heat inducible genes is not significantly increased with culture temperature between 15 and 20 °C in *C. elegans* (A). Expression of hsp20 genes is not significantly reduced under RNAi knockdown of *hsf-1* or *daf-16* in *C. elegans* at 15 °C (B and C). Expression of *hsp-16* genes is not significantly reduced under RNAi knockdown of *hsf-1* or *daf-16* in *C. elegans* at 15 °C (A and B). Mean ± SEM, Kruskal-Wallis test with a Dunn's multiple comparison test, $n \geq 3$,* = $p < 0.05$, ** = $p < 0.01$, *** = $p < 0.001$.

Figure S3.

A)

```
Gro-hsp-110.2    1 MSGIDFGNLNCYMAVARNKGIDIVENDCSLHATPACVSFGQFARMIGYGAKQQLNVNYRN
Gro-hsp-110.1    1 MSGIDFGNLNCYMAVARNKGIDIVENDCSLHATPACVSFGQFARMIGYGAKQQLNVNYRN

Gro-hsp-110.2   61 SVINFKHILGRTFADPIVQTFRHHIPCGVMGQFPDDQIGIKVSHLGTVRLLSPEQVVGCL
Gro-hsp-110.1   61 SVINFKHILGRTFADPIVQTFRHHIPCGVMGQFPDDQIGIKVSHLGTVRLLSPEQVVGCL

Gro-hsp-110.2  121 LTHLRLLLSRSLGTDVTECVINVPFFYGETQRLALLNAGRIAGLSLRLITDPAALAYCYG
Gro-hsp-110.1  121 LTHLRLLLSRSLGTDVTECVINVPFFYGETQRLALLNAGRIAGLSLRLITDPAALAYCYG

Gro-hsp-110.2  181 MYNGPSLPPPDKPSRLVAFVDAGHSSVQASLIAFNAGKAQVIAAAFDLGVGGVHFDSLLR
Gro-hsp-110.1  181 MYNGPSLPPPDKPSRLVAFVDAGHSSVQASLIAFNAGKAQVIAAAFDLGVGGVHFDSLLR

Gro-hsp-110.2  241 DHFNDNFCKRYKIDARKNARAWLRLLDECEKLKKQMSANSQSIPFSIECFMNDVDVNGSM
Gro-hsp-110.1  241 DHFNDNFCKRYKIDARKNARAWLRLLDECEKLKKQMSANSQSIPFSIECFMNDVDVNGSM

Gro-hsp-110.2  301 ERNQFERLAENLFQKVKLLLQQLVEKAKIKVGNIYDVELVGGSSRIPRLKQIVAEFFKKE
Gro-hsp-110.1  301 ERNQFERLAENLFQKVKLLLQQLVEKAKIKVGNIYDVELVGGSSRIPRLKQIVAEFFKKE

Gro-hsp-110.2  361 PKTTMNQDDAMARGCALRSAMLYPAYHMKQFAVTDPYELRVDSTTIFNEEKLTEEINVEQ
Gro-hsp-110.1  361 PKTTMNQDDAMARGCALRSAMLYPAYHMKQFAVTDPYELRVDSTTIFNEEKLTEEINVEQ

Gro-hsp-110.2  421 EMQAADLRDKQRSDAKNALEEYCFKLQHTLEDEQLCKGKVSDEERHGGIERCNAVLEWLD
Gro-hsp-110.1  421 EMQAADLRDKQRSDAKNALEEYCFKLQHTLEDEQLCKGKVSDEERHGGIERCNAVLEWLD

Gro-hsp-110.2  481 TDSETLQRKQIECRHNELDEYCRPIIGKIYAVAELD-----NKMETGASPQQSFPNDKQN
Gro-hsp-110.1  481 TESDTLQRKQIECRHNELDEYCRPIIGKIYAVAELDKNIDTNKMETGASPQQSFPNDKQK

Gro-hsp-110.2  536 VKDKKRSSDGQVEEEVQR
Gro-hsp-110.1  541 VKDKKGASDGQIEEEVQ-
```

B)

```
Sce-hsp-110        1 STPFGLDLGNNNSVLAVARNRGIDIVVNEVSNRSTPSVVGFGPKNRYLGETGKNKQTSNI
Gro-hsp-110.2      1 --MSGIDFGNLNCYMAVARNKGIDIVENDCSLHATPACVSFGQFARMIGYGAKQQLNVNY
Gro-hsp-110.1      1 --MSGIDFGNLNCYMAVARNKGIDIVENDCSLHATPACVSFGQFARMIGYGAKQQLNVNY

Sce-hsp-110       61 KNTVANLKRIIGLDYHHPDFEQESKHFTSKLV-ELDDKKTGAEVRFAGEKHVFSATQLAA
Gro-hsp-110.2     59 RNSVINFKHILGRTFADPIVQTFRHHIPCGVMGQFPDDQIGIKVSHLGTVRLLSPEQVVG
Gro-hsp-110.1     59 RNSVINFKHILGRTFADPIVQTFRHHIPCGVMGQFPDDQIGIKVSHLGTVRLLSPEQVVG

Sce-hsp-110      120 MFIDKVKDTVKQDTKANITDVCIAVPPWYTEEQRYNIADAARIAGLNPVRIVNDVTAAGV
Gro-hsp-110.2    119 CLLTHLRLLLSRSLGTDVTECVINVPFFYGETQRLALLNAGRIAGLS-LRLITDPAALAY
Gro-hsp-110.1    119 CLLTHLRLLLSRSLGTDVTECVINVPFFYGETQRLALLNAGRIAGLS-LRLITDPAALAY

Sce-hsp-110      180 SYGIFK-TDLPEGEEKPRIVAFVDIGHSSYTCSIMAFKKGQLKVLGTACDKHFGGRDFDL
Gro-hsp-110.2    178 CYGMYNGPSLPPPDKPSRLVAFVDAGHSSVQASLIAFNAGKAQVIAAAFDLGVGGVHFDS
Gro-hsp-110.1    178 CYGMYNGPSLPPPDKPSRLVAFVDAGHSSVQASLIAFNAGKAQVIAAAFDLGVGGVHFDS

Sce-hsp-110      239 AITEHFADEFKTKYKIDIRENPKAYNRILTAAEKLKKVLSANTNA-PFSVESVMNDVDVS
Gro-hsp-110.2    238 LLRDHFNDNFCKRYKIDARKNARAWLRLLDECEKLKKQMSANSQSIPFSIECFMNDVDVN
Gro-hsp-110.1    238 LLRDHFNDNFCKRYKIDARKNARAWLRLLDECEKLKKQMSANSQSIPFSIECFMNDVDVN

Sce-hsp-110      298 SQLSREELEELVKPLLERVTEPVTKALAQAKLSAEEVDFVEIIGGTTRIPTLKQSISEAF
Gro-hsp-110.2    298 GSMERNQFERLAENLFQKVKLLLQQLVEKAKIKVGNIYDVELVGGSSRIPRLKQIVAEFF
Gro-hsp-110.1    298 GSMERNQFERLAENLFQKVKLLLQQLVEKAKIKVGNIYDVELVGGSSRIPRLKQIVAEFF

Sce-hsp-110      358 GKPLSTTLNQDEAIAKGAAFICAIHSPTLRVRPFKFEDIHPYSVSYSWDKQVEDEDHMEV
Gro-hsp-110.2    358 KKEPKTTMNQDDAMARGCALRSAMLYPAYHMKQFAVTD--PY------------------
Gro-hsp-110.1    358 KKEPKTTMNQDDAMARGCALRSAMLYPAYHMKQFAVTD--PY------------------

Sce-hsp-110      418 FPAGSSFPSTKLITLNRTGDFSMAASYTDITQLPPNTPEQIANWEITGVQLPEGQDSVPV
Gro-hsp-110.2    398 ------------------------------------------------------------
Gro-hsp-110.1    398 ------------------------------------------------------------

Sce-hsp-110      478 KLKLRCDPSGLHTIEEAYTIEDIEVEEPIPLPEDAPEDAEQEFKKVTKTVKKDDLTIVAH
Gro-hsp-110.2    398 --ELRVDSTTI-------------------------------------------------
Gro-hsp-110.1    398 --ELRVDSTTI-------------------------------------------------

Sce-hsp-110      538 TFGLDAKKLNELIEKENEMLAQDKLVAETEDRKNTLEEYIYTLRGKLEEEYAPFASDAEK
Gro-hsp-110.2    407 ---FNEEKLTEEINVEQEMQAADLRDKQRSDAKNALEEYCFKLQHTLEDEQLCKGKVSDE
Gro-hsp-110.1    407 ---FNEEKLTEEINVEQEMQAADLRDKQRSDAKNALEEYCFKLQHTLEDEQLCKGKVSDE

Sce-hsp-110      598 TKLQGM--LNKAEEWLYDEGFDSIKAKYI-AKYEELASLGNIIRGRYLAKEE--------
Gro-hsp-110.2    464 ERHGGIERCNAVLEWL-DTDSETLQRKQIECRHNELDEYCRPIIGKIYAVAELD-----N
Gro-hsp-110.1    464 ERHGGIERCNAVLEWL-DTESDTLQRKQIECRHNELDEYCRPIIGKIYAVAELDKNIDTN

Sce-hsp-110      647 -------------EKKQAIRSKQEA-----------
Gro-hsp-110.2    518 KMETGASPQQSFPNDKQNVKDKKRSSDGQVEEEVQR
Gro-hsp-110.1    523 KMETGASPQQSFPNDKQKVKDKKGASDGQIEEEVQ-
```

Figure S3. Gene function is conserved in the two *hsp-110* paralogues in *G. rostochiensis*. CLUSTAL W multiple sequence alignments for the two *hsp-110* translated transcripts from *G. rostochiensis* showing non conservative mutations at residues 517, 540, 546 and 547 (A) and with *Saccharomyces cerevisae* (B) showing that non conservative mutations occur within the flexible 44 bp C-terminal region (Shaner, et al. 2004).

Figure S4

A)

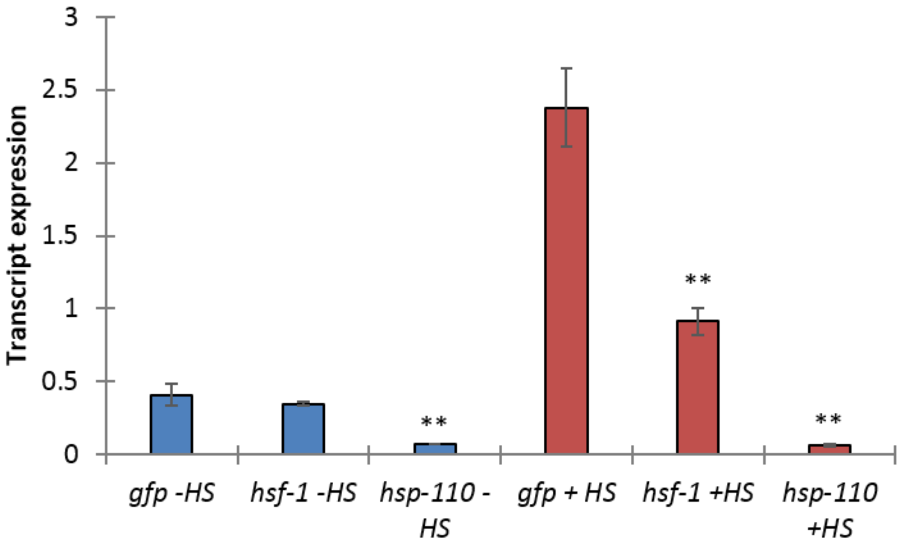

B)

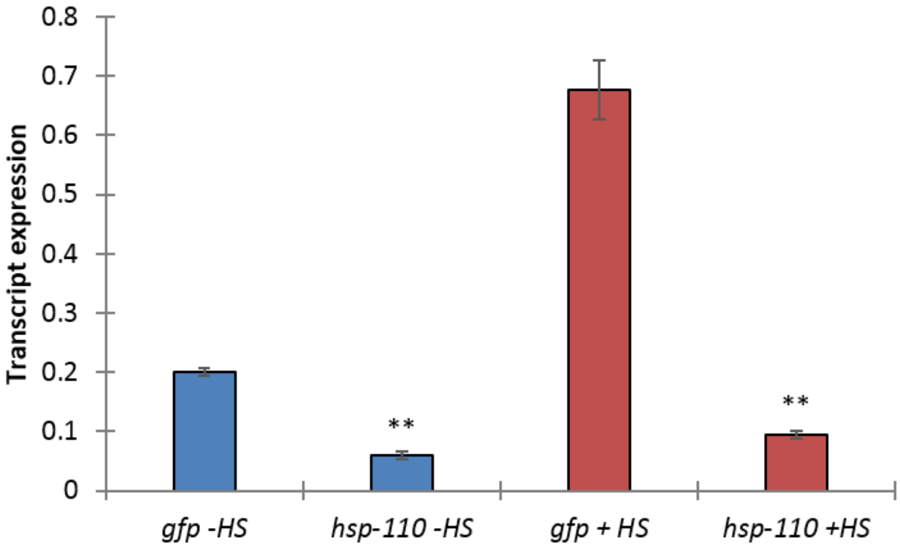

Figure S4. ***hsf-1* is required for normal induction of *hsp-110* following heat stress in *C. elegans*.** Expression of *hsp-110* was significantly reduced by ~ 62 and ~ 97 % with heat stress (35 °C for 1 hr) under RNAi knockdown of *hsf-1* and *hsp-110* in *C. elegans* respectively. Expression of *hsp-110* was also significantly reduced by ~83 % without heat stress (A). Expression of *hsp-110* was significantly reduced by ~ 86 % with heat stress and ~ 71 % without heat stress under RNAi knockdown of *hsp-110* in *G. rostochiensis* (B). Mean ± SEM, Kruskal-Wallis test with a Dunn's multiple comparison test and unpaired two-tailed Mann-Whitney test, n = 5, * = $p < 0.05$, ** = $p < 0.01$, *** = $p < 0.001$.

Oligo sequences for qPCR analysis

Gpa-act-1 F: CTT CTT GGG CAT GGA GTC GG

Gpa-act-1 R: AAT GCC CGG GTA CAT CGT C

Gpa-cdc-42 F: GCA TCA TTG TAC GAA GAC TCC A

Gpa-cdc-42 R: TGG GCT TCA TTT TGT TCT TTG C

Gpa-daf-21 F: TCC TTG TTG CAT TGT CAC CG

Gpa-daf-21 R: TAA TGA TCG AGT GGT CCG GG

Gpa-dnj-13 F: CAT CAA AGA CAA GCC GCA CT

Gpa-dnj-13 R: TTC AAT GGA ATC GTG TCG CC

Gpa-gly-8 F: CAT CGA CTC CAG CCT GTA CA

Gpa-gly-8 R: CCT TCA GCT TCA GAC GTT CG

Gpa-hsp-1 F: TCA GAC GTT CAC CAC CTA CT

Gpa-hsp-1 R: GAC TTC AAT TTG AGG CAC ACC

Gpa-hsp-16.1 F: CAA CGT CGA ACT GAA GGG C

Gpa-hsp-16.1 R: GGA ATG AGC ACA CGA CGA CA

Gpa-hsp-16.2 F: CAC CGC GAG CAG AAC CAC

Gpa-hsp-16.2 R: TGG TCT TCA TGC CGG TGA TG

Gpa-hsp-16.3 F: GAG CTG AAG GAC AAT GAG ATC A

Gpa-hsp-16.3 R: CCC GTC CAG CTC GCA TTT

Gpa-hsp-16.4 F: CAG AAG CAG GGC GAA TCG

Gpa-hsp-16.4 R: TGG GAA TGG AGC GCT TCT

Gpa-hsp-16.5 F: GTG AAC ACC ACC ACG CAA CA

Gpa-hsp-16.5 R: TCG CCA TGC AGC TCA ACA G

Gpa-hsp-16.6 F: GAC CCG TTC CGT CCC ATG

Gpa-hsp-16.6 R: GTG AAG TTT CCC AAG TCG TCA

Gpa-hsp-110 F: ATT GTC GAC CGA TTA TTG GAA AG

Gpa-hsp-110 R: CAA TTT GCC CAT CCG ACG C

Gpa-kin-20 F: AGC TGC TAT TGG AA GTG GT

Gpa-kin-20 R: AAC TCA TCG TCT CCA TCG CT

Gpa-mnk-1 F: ACA GCC ACC AAT TTG TCA CG

Gpa-mnk-1 R: GAA ATG CGC CAA GTT GTG TC

Gpa-ndk-1 F: CGG ATC ACT ACA TTG GCT GCT

Gpa-ndk-1 R: AAT TTT GTC GGG AGT GGC AC

GPLIN_001642000 F: GTG ACG TCC TCA ATG AGC G

GPLIN_001642000 R: GAC ATT CGG CTC AAG GAG GA

Gro-act-1 F: CTT CTT GGG CAT GGA GTC GG

Gro-act-1 R: AAT GCC CGG GTA CAT CGT C

Gro-cdc-42 F: GCA TCA TTG TAC GAA GAC TCC A

Gro-cdc-42 R: TGG GCT TCA TTT TGT TCT TTG C

Gro-daf-21 F: TCC TTG TTG CAT TGT CAC CG

Gro-daf-21 R: TAA TGA TCG AGT GGT CCG GG

Gro-dnj-13 F: CGG GTT GGA AGA GCG GTA

Gro-dnj-13 R: GAG CCG TCT CGT CTG AAC TT

Gro-gly-8 F: ACT CCA GCC TGT ACA ACT CG

Gro-gly-8 R: GCA GTT CAG CTT CTC CTT CAG

Gro-hsp-1 F: ACA AAA TCA GTG CCG ACG AC

Gro-hsp-1 R: GAC AGC CTC CAA CTC CTT CT

Gro-hsp-16.1 F: GTG CAC CGC CAG TTC TGT

Gro-hsp-16.1 R: CAA TGG GAA TGG AGC GCT TC

Gro-hsp-16.2 F: GAG GAG GTC AAG GTC GAA C

Gro-hsp-16.2 R: GGT CTT CAT GCC GGT GAT GT

Gro-hsp-16.3 F: TGA ACT GAA GGA CAA TGA GAT CA

Gro-hsp-16.3 R: GGA ATG AGC ACA CGA CGA CT

Gro-hsp-16.4 F: CCA CGT CGC ACA GCT GAC

Gro-hsp-16.4 R: GAT GCA CCG ACT CGC CTT

Gro-hsp-16.5 F: GGC CTA CCG TTT CGA TGT G

Gro-hsp-16.5 R: GCA CGG ACA GGC CTT CTT

Gro-hsp-16.6 F: GAC CCG TTC CGT CCC ATG

Gro-hsp-16.6 R: GTG AAG TTT CCC AAG TCG TCA

Gro-hsp-110 F: ATT GAG CGC TCG GGA CTG

Gro-hsp-110 R: GTT GCT TGG CGG AAT TTT GC

Gro-hsp-110.1 F: ATT GTC GAC CGA TTA TTG GAA AG

Gro-hsp-110.1 R: CAA TTT GCC CAT CCG ACG C

Gro-hsp-110.2 F: GGA TGA ATA TTG TCG ACC GAT CA

Gro-hsp-110.2 R: CTT GCC CAT CCG ATG ACC TC

Gro-kin-20 F: GTT CAG ACA ACG AGC TGC TG

Gro-kin-20 R: GGT CAG CTC ATC GTC TCC AT

Gro-mnk-1 F: GACACAACTTGGCGCATTTC

Gro-mnk-1 R: CATCATGTTCAGGCCGTTCA

Gro-ndk-1 F: TCC AAT GAG CCC TAC TTC CC

Gro-ndk-1 R: AAT TTT GTC GGG AGT GGC AC

GROS_g03258 F: GAC ATT CGG CTC AAG GAG GA

GROS_g03258 R: TCC TCA ATG AGC GGT CCT TC

Cel-act-1 F: ACC CAG GAA TTG CTG ATC GT

Cel-act-1 R: TCT GTT GGA AGG TGG AGA GG

Cel-cdc-42 F: TGT TTG CTT CTC CGT GGT TG

Cel-cdc-42 R: TCT CGA GCA TTC CTG GAT CA

Cel-hsp-16.1 F: AGA TAT GGC TCA GAT GGA ACG T

Cel-hsp-16.1 R: ATC TTC TGG CTT GAA CTG CG

Cel-hsp-16.2 F: TGG TCG TAC GCT ATC AAT CC

Cel-hsp-16.2 R: TCT TTG GCG CTT CAA TCG AA

Cel-hsp-16.41 F: TGA TGA ATC CAA GTT TTC GGT TC

Cel-hsp-16.41 R: CGT TTC AAG TAT CCA TGT TCC GA

Cel-hsp-16.48 F: TGC TCC GTT CTC CAT TTT CTG

Cel-hsp-16.48 R: ACA ATC TCT CCA ATA TTG TCG GA

Cel-hsp-110 F: ACG ATG AAT CAA GAC GAG GC

Cel-hsp-110 R: CTG TTC CAG CTG AGA CGA AT

Cel-hsf-1 F: CGT GCG ATG CGA GAA AAG G

Cel-hsf-1 R: CTG TTG GCG AGC ATG TTG TT

Cel-kin-20 F: GGA ACT GCA AGA TAC GCC AG

Cel-kin-20 R: CAG CTT TTA GTC CCT GCC AC

Cel-mnk-1 F: GTG GGG TAT GAT CTC CGA GG

Cel-mnk-1 R: GAG CCA GCG ATG AGA GAG AA

Cel-ndk-1: CGT TTT GAA GAG CGC GGA TA

Cel-ndk-1: CTC GAT GAG GGA TGG GAA GA

Oligo sequences for cloning and sequencing of *Globodera* hsp-110 promoter and gene

Globodera hsp-110 gDNA cloning F: TTG GAC TTC GCT TTT CCC TC

Globodera hsp-110 gDNA cloning R: GCT TTC TTT ACC ACC AGT CTC C

Gpa-hsp-110 gDNA sequencing F1: TCA AGG TCA TCG AGA AAC ACA

Gpa-hsp-110 gDNA sequencing F2: TTT CGG CAC CAT ATC CCT TG

Gpa-hsp-110 gDNA sequencing F3, TTT CGG CAC CAT ATC CCT TG

Gpa-hsp-110 gDNA sequencing R1: ATC GTC GTC TTG GGC TCT TT

Gpa-hsp-110 gDNA sequencing R2: GGC ATC GAC AAA GGC AAC

Gpa-hsp-110 gDNA sequencing R3, GGC ATC GAC AAA GGC AAC

Gro-hsp-110.1 gDNA sequencing F1: TCA AGG TCA TCG AGA AAC ACA

Gro-hsp-110.1 gDNA sequencing F2: TTT CGG CAC CAT ATC CCT TG

Gro-hsp-110.1 gDNA sequencing F3, TTT CGG CAC CAT ATC CCT TG

Gro-hsp-110.1 gDNA sequencing R1: ATC GTC GTC TTG GGC TCT TT

Gro-hsp-110.1 gDNA sequencing R2: GGC ATC GAC AAA GGC AAC

Gro-hsp-110.1 gDNA sequencing R3, GGC ATC GAC AAA GGC AAC

Gro-hsp-110.2 gDNA sequencing F1: GGA TCC GCG GCC ACT TTT AC

Gro-hsp-110.2 gDNA sequencing F2: GCG GAA CAA AGG AAT CGG TT

Gro-hsp-110.2 gDNA sequencing F3, GCG GAA CAA AGG AAT CGG TT

Gro-hsp-110.2 gDNA sequencing R1: GCT TTT CAA ATG CCT TTC CCA

Gro-hsp-110.2 gDNA sequencing R2: CCG GCA TCA ACA AAG GCA A

Gro-hsp-110.2 gDNA sequencing R3, CCG GCA TCA ACA AAG GCA A

Oligo sequences for preparation of pl4440 constructs

Cel-hsp-110 F: ataggtaccAGGCAATGGAAGTGGATGGA

Cel-hsp-110 R: ataagatctGCATCTTCTCCCTCGTCGTA

Cel-hsf-1 F: ataggtaccGCTCACGTGAACAATCTCCA

Cel-hsf-1 R: ataagatctTGTTCACAAATCCTCCCGAA

Cel-daf-16 F: ataggtaccCGACTACAAAGGCTCAACTCG

Cel-daf-16 R: ataagatctACTTGGAATTGCTGGAACCG

Gro-hsp-110 F: ataggtaccGAATCAGGACGACGCAATGG

Gro-hsp-110 R: ataagatctCGCGTTGCACCTTTCGAT

Gro-hsf-1 F: ataggtaccATGATCAACAGCTCGCATCG

Gro-hsf-1 R: ataagatctGCGGTCGTTGTTGAGGATAC

gfp F: ataggtaccTTTTCAAGAGTGCCATGCCC

gfp R: ataagatctTGGTAAAAGGACAGGGCCAT
